# Competency-based pre-service education for clinical psychology training in low- and middle-income countries: Case study of Makerere University in Uganda

**DOI:** 10.3389/fpsyg.2022.924683

**Published:** 2022-10-10

**Authors:** Benjamin Alipanga, Brandon A. Kohrt

**Affiliations:** ^1^Department of Mental Health and Community Psychology, Makerere University, Kampala, Uganda; ^2^Division of Global Health, Department of Psychiatry and Behavioral Sciences, George Washington University, Washington, DC, United States

**Keywords:** competency, competency-based, curriculum, practicum/internship, EQUIP, training

## Abstract

Reducing the global treatment gap for mental health conditions in low- and middle-income countries (LMICs) requires not only an expansion of clinical psychology training but also assuring that graduates of these programs have the competency to effectively and safely deliver psychological interventions. Clinical psychology training programs in LMICs require standardized tools and guidance to evaluate competency. The World Health Organization (WHO) and UNICEF developed the “Ensuring Quality in Psychological Support” (EQUIP) platform to facilitate competency-based training in psychosocial support, psychological treatments, and foundational helping skills, with an initial focus on in-service training for non-specialists. Our goal was to design the first application of EQUIP to implement competency-based training into pre-service education for clinical psychology trainees. With Makerere University in Uganda as a case study, we outline an approach to develop, implement, and evaluate a competency-based curriculum that includes seven steps: (1) Identify core clinical psychology competencies; (2) Identify evaluation methods appropriate to each competency; (3) Determine when competency evaluations will be integrated in the curriculum, who will evaluate competency, and how results will be used; (4) Train faculty in competency-based education including conducting competency assessments and giving competency-based feedback; (5) Pilot test and evaluate the competency-based education strategy with faculty and students; (6) Modify and implement the competency-based education strategy based on pilot results; and (7) Implement ongoing evaluation of the competency-based curriculum with continuous quality improvement. This approach will be formally evaluated and established as a foundation for pre-service training in other low-resource settings.

## Background and rationale

Reducing the current global mental health treatment gap requires not only expanding the specialist and non-specialist workforce but also assuring that this workforce is competent to offer effective services. Uganda exemplifies the challenges for low- and middle-income countries (LMICs) which experience myriad war-related traumas, chronic poverty, and high rates of infectious disease with associated early mortality. Training a mental health workforce in Uganda, as with other LMICs, is beset with various challenges including unclear contents of curriculum, unspecified teaching methods, lack of, or inadequate supervision, un-standardized evaluation methods, and poor access to information resources. Nsereko (2015) described mental-health training in Uganda as more comparable to liberal arts training than to a professional degree; for example, there is a lack of consideration for the numbers of trainees per trainer, lack of skill-based counselor trainers’ qualification, and failure to effectively build skills during practicums and internships due to poorly resourced placement sites, as well as lack of professionals with the time available and expertise in supervision. These challenges translate into ineffective mental health care as students are poorly trained and may not be competent and effective service providers. To overcome some of the challenges of mental-health training, there is a growing attention to competency-based education, particularly in the field of professional psychology (Roberts et al., 2005; Rodolfa et al., 2005; Mills et al., 2020).

Mills et al. (2020) defined competency as having requisite knowledge, attitude and skills for a particular field: knowledge is an unobservable attribute of competence and can be inferred through performance or specific testing, skill is a specific cognitive or motor ability (observable and unobservable) typically developed through training and practice, and attitude is a person’s feelings, values and beliefs, which influence behavior and performance of tasks and can be observed. In competency-based training, the competencies form the basis for identifying the objectives of training, planning training activities, and evaluation process (Kaslow, 2004) and thus facilitate learning, assessment, and supervision. Mental-health professionals who demonstrate competence are more likely to deliver psychological treatments that are effective and safe for their clients (McHugh and Barlow, 2010).

Because of a recognition that there were not standardized methods that could feasibly and consistently be applied in LMICs, the World Health Organization (WHO) and UNICEF developed the Ensuring Quality in Psychological Support (EQUIP) platform (Kohrt et al., 2020). The EQUIP platform is a digital tool, available in online and offline formats, and associated resources to implement competency-based training. Competency assessment tools are available for mental healthcare with adults, children and adolescents, and group-based formats (Kohrt et al., 2015a; Jordans et al., 2021; Pedersen et al., 2021). There are also treatment package competencies for WHO manualized interventions such as Problem Management Plus (PM+) and the Thinking Healthy Programme (THP), as well as specific techniques for diverse therapy classes including cognitive, problem-solving, interpersonal, trauma-related, motivational enhancement, and stress management techniques (Pedersen et al., 2020, 2021). The platform includes guidance on conducting role plays for competency-based training, incorporating competency-based feedback into training, and a data-tracking and data-visualization feature to monitor changes in competency over time. EQUIP has been evaluated in diverse LMIC settings when training non-specialists.

However, the EQUIP platform has not yet been systematically integrated into pre-service training of mental-health professionals. Formal training institutions are best placed to ensure sustainability of training, and competency maintenance for effective mental-health care. Moreover, if specialist pre-service programs integrate resources such as EQUIP, this prepares their graduates to conduct competency-based approaches in their future training of non-specialists, i.e., it has a potential cascade effect. The current initiative at Makerere University in Uganda therefore aims to develop a competency-based curriculum for pre-service training of mental-health professionals in formal training institutions using the tools and resources in the EQUIP platform.

## Current masters of science clinical psychology training curriculum of Makerere University

The School of Psychology at Makerere University in Kampala, Uganda, offers a Master’s of Science degree in clinical psychology. The courses are taught through interactive learning approaches including tutorials, field work, case studies, small group and class discussions, and self-study (see Supplementary Table 1). Students on practicums and internships are trained through actual work with patients under supervision of a placement site supervisor and a university supervisor. Students attend weekly class consultation and supervision with the course coordinator to discuss their practicum or internship experiences.

Evaluation, both formative and summative, is based on the learning methods, e.g., case reports based on patients worked with during practicum, class presentations on pre-assigned topics, self-study, and assigned readings. Small groups of three to five students are supervised and assessed for knowledge, attitudes and skills using an assessment guide, and a report is given to the university supervisor. Tests in the form of “true/false” response, multiple choice questions (MCQs), and case scenarios may be given by specific course instructors. Students on practicum placement are supervised by site supervisors who give assessment reports based on the same assessment guide used by university supervisors for small group supervision; thus, for every student there are at least two scores (one from the practicum supervisor and one from the university supervisor) on the domains of knowledge, attitude, and skills. Finally, end of semester examination is administered as a summative evaluation. Certification requires a student to pass all the courses including the final examination at a minimum 60% mark obtained cumulatively from the different assessment methods described above. Unfortunately, there is no consistency across courses and instructors with regard to what skills are prioritized and how they are evaluated. The choice of assessment format, evaluation method, and grading are not standardized and depend on the individual course lecturer. Moreover, there are not curriculum-wide strategies to address gaps in clinical skills. Furthermore, the 60% minimum requirement does not differentiate among knowledge and skill-based assessments, therefore a trainee could do well on all knowledge assessments but poorly on skill-based demonstrations and still successfully complete the master’s program. Based on these concerns, we explored developing a competency-based curriculum for Makerere University, which integrates the EQUIP platform resources and assessments.

## Competency-based curriculum

A competency-based framework (Table 1) organizes how trainees acquire knowledge, skills, and attitudes, which together form the desired outcome of the training program and are practice-driven ultimately to achieve clinical behavior change (Anema and McCoy, 2010; Boland, 1998). In this framework, the training setting becomes an intentional, structured environment for context-specific learning, skill development, and assessment (Wittmann-Price and Karen, 2012). The learning resources for each course are aligned with the assessments that evaluate the competencies (Johnstone and Soares, 2014) so that the competency, the content being learned, and the assessment all have a well-defined relationship (Shinners and Graebe, 2019). The end of the semester becomes the “outcome point” (Falender and Shafranske, 2004) measured through summative evaluation. The competency-based curriculum represents a more scientific and systematic approach to tracking training outcomes (Nelson, 2007).

**Table 1:** Competency-based curriculum.

	Month	Coursework	Practical skills training	Assessment points	Use competency results to:
Year 1	September October November December	Semester 1			
1		Theoretical Models in Clinical Psychology	*Practicum exposure* (Butabika NMHRH) 1-Counseling skills 2-Ethical attitude and behavior 3-Professionalism, 4-Reflective practice.	1. Before practicum exposure *Formats*: Role plays, video recordings, audio recordings, transcripts.	Give results to practicum supervisor so they can assess trainees’ base-line competency levels to inform training plan (FHS). Record a baseline of competency to track trainees’ progress and performance across training.
		Professional and Ethical Studies			
		Adult psychopathology			
		Advanced Research Methods			
		Semester 2			
	February March April May	Psychological Assessment and Interviewing		2. After practicum exposure Formats: Role plays, video and audio recordings, transcripts.	To confirm minimum competency levels achieved and compare pre- and post-assessments to examine effectiveness of training program. To focus skill development during practicum. (FHS)
		Family and Marital Psychotherapy			
		Adult and Child Psychotherapies			
		Advanced Statistics			
		Psychological Assessment and Interviewing			
		Recess Term			
2	May June July	Dissertation/ Clinical Proposal Presentation	*Practicum* (Butabika NMHRH) 1-Counseling, 2-Clinical -Assessment, 3-Case conceptualization 4-Intervention.	3. Before practicum *Formats*: Role-plays, live observations, video recordings, audio recordings, supervisor reports.	To track progress Inform any needed adjustments to training activities. Measure maintenance or drift in skills.
		Practicum/ Internship			
	August	Break			
Year 2		Semester 3			
	September October November December	Global Mental Health		4. After practicum *Formats*: Role plays, video and audio recordings, transcripts.	To confirm minimum competency levels achieved and compare pre- and post-assessments to examine effectiveness of training program. Track improvement and maintenance of competencies over time. To focus skill development during internship. (Treatment specific: IPT, PM+)
		Health Psychology			
		Child and Adolescent Psychotherapy			
		Psychopharmacology			
		Dissertation/Case write Up I			
		Semester 4			
3	February March April May	Cultural Issues in Psychotherapy	*Internship* (Butabika NMHRH) 1-Clinical Assessment, 2-Case conceptualization 3-Intervention 4-Supervision	5. Before internship *Formats*: Live observations, video recordings, audio recordings, supervisor reports	Track progress during internship. (Treatment specific: IPT)
		Internship			
		Dissertation/Case write Up II		6. After internship *Formats*: Live observations, video recordings, audio recordings, supervisor reports.	Evaluate trainees to confirm minimum competency levels are met. Compare pre- and post-assessments to examine effectiveness of training program. Inform remediation needs and activities. Determine whether or not ready for work in the real world and determine giving certificate. (Treatment specific: IPT)
		Cultural Issues in Psychotherapy			
		*Electives*			
		Psychosocial Care of the Dying and Bereaved			
		Gender Issues In Psychotherapy			

We propose seven steps to develop, implement, and evaluate a competency-based curriculum in pre-service training:

1. Identify core competencies of the curriculum.
2. Identify methods of evaluation appropriate to each competency.
3. Determine when competency evaluations will be integrated in the curriculum, who will evaluate competency, and how results will be used.
4. Train faculty in competency-based education including training, competency assessments, and giving competency-based feedback.
5. Pilot test and evaluate the competency-based education strategy with faculty and students.
6. Modify and implement the competency-based education strategy based on pilot results.
7. Implement ongoing evaluation of the competency-based curriculum with continuous quality improvement.

Below we describe each of these seven steps as they relate to planned activities for the Masters of Science degree in clinical psychology.

### Identify core competencies of competency-based curriculum

Professional psychology competencies are broadly divided into two categories: foundational and functional competencies. Foundational competencies, also known as common factors, are the mental-health care provider’s skills that relate to building a warm, trustworthy relationship between client and the therapist (Barth et al., 2012; Laska et al., 2014). These factors serve as the foundation for the normal functions of a psychologist (Kaslow et al., 2009). Some foundational skills identified in evidence-based interventions delivered in LMICs include promoting hope and realistic expectancy for change; collaborative goal setting; explaining and assuring confidentiality; family engagement; giving praise; eliciting feedback; psychoeducation; normalization and validation of emotions; rapport building and self-disclosure; assessment of harm; empathy; non-verbal communication; and verbal communication (Pedersen et al., 2020). Foundational competencies are assumed to be universal for the delivery of any effective treatment and play important role in generating effective outcomes (Mulder et al., 2017). Competent use of these skills has been associated with improved treatment outcomes for people accessing mental-health and for the whole range of health services (Han and Pappas, 2018; Mills et al., 2020). Functional competencies are the specific tasks and functions carried out by psychologists, and include assessment, intervention, consultation, and supervision (Rodolfa et al., 2013).

Efforts to define and assess competencies to assure safe and appropriate psychological practice have been ongoing (Rubin et al., 2007; Roth and Pilling, 2008; Gonsalvez et al., 2021). Among different available frameworks, we selected the Cube Model of professional psychological competencies (Fouad et al., 2009; see Table 2), which includes (1) counseling, (2) clinical assessment, (3) case-conceptualization, (4) intervention, (5) ethical attitude and behavior, (6) scientist-practitioner, (7) Professionalism, (8) psychological testing, (9) reflective practice, and (10) supervision competencies.

**Table 2:** Clinical psychology competencies.

Competency	Description	Ideal behavior
1. Counseling	Ability to show empathic understanding, to apply basic counseling techniques, and collaborative goal formulation with clients.	(a) Applies basic counseling techniques appropriately including clarification, paraphrase and summarizing responses. (b) Forms and communicates an empathic understanding to clients, careers, and significant others. (c) Formulates client goals in a collaborative manner. (d) Demonstrates accurate empathy in complex situations where affect is covert, controlled or denied.
2. Clinical Assessment	Ability to perform adequate assessments in a time efficient and in a personally/socio-culturally sensitive manner; to appropriately prioritize issues, and assess risk.	(a) Demonstrates knowledge of psychopathology and diagnostic criteria for clients seen at the placement. (b) Demonstrates a systematic and logical sequence of questioning during the clinical assessment interview. (c) Skillful and efficient in conducting a clinical assessment, including a mental state examination. (d) Undertakes clinical assessments in an interpersonally engaging and in a socio-culturally sensitive manner.
3. Case Conceptualization	Ability to appropriately integrate information from multiple sources to inform appropriate case conceptualizations, diagnoses, and treatment plans.	(a) Makes appropriate use of diagnostic frameworks (e.g., DSM5) to arrive at correct diagnoses and differential diagnoses. (b) Draws upon different psychological theories and approaches to derive a meaningful case conceptualization. (c) Integrates cultural knowledge into case conceptualization. (d) Integrates assessment and other information into realistic treatment plans.
4. Intervention	Ability to skillfully implement appropriate, empirically supported treatment interventions; monitor treatment progress and outcomes.	(a) Demonstrates knowledge of principles and procedures of relevant interventions. (b) Demonstrates effective application of theoretical knowledge of evidence-based treatment methods (e.g. CBT, IPT, MI). (c) Implements interventions relevant to the needs of the client. (d) Demonstrates flexibility and responsiveness in the application of treatments and/or in the implementation of scheduled programs. (e) Efficiently conducts evidence-based treatment approaches (e.g. CBT, IPT, MI). Fluently transitions between elements/techniques. (f) Overcomes common difficulties in therapy through skillful interviewing to maintain therapy direction and progress. (g) Uses appropriate measures to regularly monitor treatment progress and outcomes.
5. Ethical Attitude and Behavior	Knowledge of ethical/professional codes, standards and guidelines, and commitment to their application. Ability to maintain appropriate and respectful boundaries and seek consultation on ethical issues.	(a) Demonstrates knowledge of ethical/professional codes, standards and guidelines. (b) Recognizes ethical and legal issues that arise across the range of professional activities, and demonstrates good discernment and judgment in these situations. (c) Acknowledges the limits of one’s competence and makes appropriate referrals when required. (d) Demonstrates commitment to ethical practice across a range of clinical situations.
6. Scientist Practitioner	Knowledge of theoretical and research evidence related to diagnosis, assessment and intervention. Able to show respect for scientific methods and empirical evidence and commitment to their application to clinical practice.	(a) Demonstrates knowledge of theoretical and research evidence related to assessment, diagnosis, case conceptualization and treatment, and to intervention monitoring and evaluation of interventions. (b) Demonstrates the ability to critically analyze and evaluate the empirical literature. (c) Demonstrates respect for, and use of, the scientific method in clinical practice. (d) Demonstrates systematic and habitual application of scientific principles (e.g., hypothesis testing) to assessment, diagnosis, case conceptualization and treatment, and to intervention monitoring and evaluation of interventions.
7. Professionalism	Effective organization and time management. Clear and professional expressive skills, professional dress and demeanor. Good interactional skills with colleagues and other professionals.	(a) Demonstrates responsibility and accountability, reliably and punctually attending client appointments and work-related activities. (b) Demonstrates an organized, disciplined, and timely approach to maintaining case notes and records. (c) Effectively prioritizes competing tasks. (d) Demonstrates concern for the welfare of others including the profession, organization and community, and shows respect for cultural values and diversity. (e) Clearly and effectively communicates in verbal, non-verbal and written forms for a range of purposes. (f) Conducts self professionally in dress and demeanor. (g) Works collaboratively with colleagues across a range of disciplines. (h) Copes professionally with disapproval and criticism, and works constructively toward resolution of interpersonal conflicts at work. (i) Demonstrates progress in developing an integrated sense of self as a professional psychologist.
8. Psychological Testing	Able to apply knowledge to correctly select, administer, score and interpret common psychometric tests, and to generate psychometric reports. Knowledge of psychometric issues and testing theory.	(a) Correctly administers and score common/core psychological tests. (b) Demonstrates knowledge of psychometric issues, testing theory, and bases of assessment methods. (c) Interprets and integrates information in accordance with psychometric principles. (d) Demonstrates ability to write psychological test reports that are clear, accurate, and tailored appropriately to the user.
9. Reflective Practice	Self-care, self-awareness and reflectivity, reflection on own emotions, beliefs, values and behavior and their effect on others. Appropriately self corrects.	(a) Demonstrates problem-solving ability, organized reasoning, intellectual curiosity and flexibility. (b) Demonstrates affect tolerance, understanding of interpersonal conflict, tolerance of ambiguity and uncertainty. (c) Demonstrates consideration of the way in which personal issues and concerns impact on one's professional practice. (d) Effectively uses observation and feedback including supervision to hone reflection skills. (e) Actively reflects on ways in which others' cross-cultural values and perspectives influence one's own responses and vice versa. (f) Accurately assesses own strengths and weaknesses and level of competence and plans necessary learning to address gap. (g) Demonstrates appropriate and timely care of personal health and wellbeing to ensure effective professional functioning.
10. Supervision	Able to show good preparation and collaboration within supervision, openness to and effective use of feedback.	(a) Demonstrates adequate preparation for supervision. (b) Seeks and accepts supervisory input, including direction. (c) Appropriately balances autonomy and dependency needs.

### Identify methods of evaluation appropriate to each competency

Drawing upon Miller’s assessment strategies for the hierarchy of clinical skills (Miller, 1990), Kohrt and colleagues (Ottman et al., 2020) outlined approaches to assess knowledge, attitudes, and skills of service providers using conventional measurements as well as role-play based skills assessments. Measurement of conceptual knowledge is assessed through “true/false” questions and MCQs. Knowledge of how to apply theory is measured through decision-making questions following clinical vignettes. Ability to apply skills is measured through standardized role-plays and standardized patients. Lastly, how therapists apply skills in practice (therapist quality) is assessed through standardized rating of treatment sessions. These methods will be replicated in Makerere’s program and used in training and assessment of pre-service psychology students. EQUIP tools relevant for Makerere’s clinical psychology program include the Enhancing Assessment of Common Therapeutic factors (ENACT) for measuring adult professional psychology skills; Working with children—Assessment of Competencies Tool (WeACT) for assessing service providers’ competencies in helping children and adolescents; the GroupACT used for group facilitation assessment; and treatment specific tools for Interpersonal Psychotherapy (IPT) and Problem Management plus (PM+). These tools have been used in various settings for low-intensity psychological interventions (Kohrt et al., 2015a, 2018; Pedersen et al., 2021) and found to be effective for training, supervision and assessment of competencies (Singla et al., 2017; Ottman et al., 2020).

Role plays will be used with standardized clients and scored by trained raters, and the results will form the basis of scores to reflect competency attainment. All of the EQUIP assessment tools are structured in a format to allow for easy scoring and delivery of competency-based feedback. The ENACT and other tools have a scoring system consisting of skill categories with a short description of observable behaviors associated to a level for each behavior. The score categories are unhelpful or potentially harmful behaviors (Level 1); lack of harmful behaviors with demonstration of some basic helping behaviors (Level 2); demonstration of all basic helping behaviors (Level 3); and demonstration of all basic helping behaviors plus some advanced helping behaviors (Level 4). Trained raters observe and score behaviors demonstrated by the trainee in structured role plays. The results are used to guide decision-making about the need for improvement and remediation of training and supervision. Feedback tailored to Level 1 is reducing harmful behaviors; for Level 2, the goal is to learn and demonstrate more basic skills; for Level 3, feedback reinforces use of the basic skills and encourages integration of advanced skills; Level 4 emphasizes affirmation of the broad range of skills demonstrated. The system allows for continuous improvement in training and supervision as needed (Kohrt et al., 2018). In the Makerere curriculum, MCQs and “true/false” questions will be administered to supplement the observable skill ratings. This is helpful to determine if a lack of knowledge underlies the gap in skills.

### Determine when competency evaluations will be integrated in the curriculum, who will evaluate competencies, and how results will be used

The competency evaluation will be carried out by university faculty and placement site supervisors, all are qualified mental-health professionals, who will receive training on the EQUIP platform including how to conduct competency assessments and how to give competency-based feedback. We propose six assessment points (see Table 3) during the two-year training period for clinical psychology students including: before initial clinical exposure, after initial clinical exposure, before practicum, after practicum, before internship, and after internship.

**Table 3:** Competency assessment schedule.

Assessment #	When conducted	Competencies (e.g., foundational skills, IPT)	Format (structured role play, or client observation)	Who acts as client	Who conducts rating	Who receives results	How are results used	What modifications are needed for implemen-tation
**1**	Before practicum exposure	*Fundamental* 1-Counseling skills 2-Ethical attitude and behavior 3-Professionalism 4-Reflective practice.	*Modality*: Standardized role plays with mock group members *Formats*: Live observations, video recordings, audio recordings, transcripts	Trainers and students	University rators and supervisors	Students, Practicum coordinator and university supervisors	Assess trainees’ competency levels to inform training plan; Record a baseline of competency to track trainees’ progress and performance across training.	Training and assessment skills to supervisors and program coordinators
**2**	After practicum exposure	*Fundamental* 1-Counseling skills 2-Ethical attitude and behavior 3-Professionalism 4-Reflective practice.	*Modality*: Standardized role plays with mock group members *Formats*: Live observations, video recordings, audio recordings, transcripts	Trainers and students	University rators and supervisors	Students, practicum coordinator and university supervisors	Assess trainees’ competency levels to inform training plan; Record a baseline of competency to track trainees’ progress and Performance across training.	Training and assessment skills to supervisors and program coordinators
**3**	Before practicum	*Functional* 1-Counseling skills 2-Clinical -Assessment, 3-Case conceptualization 4-Intervention	Modality: Periodic role-plays; single competency role plays Formats: Live observations, video recordings, audio recordings, transcripts	Trainers and students	University rators and Supervisors	Students, practicum coordinator and university supervisors	To track and record trainees’ progress during training; Measure maintenance or drift in skills; Inform any needed adjustments to training activities.	
**4**	After practicum	*Functional* 1-Counseling skills 2-Clinical -Assessment 3-Case conceptualization 4-Intervention	*Modality*: Standardized role plays with mock group members *Formats*: Live observations, video recordings, audio recordings, transcripts	Students	University rators and Supervisors	Students, practicum coordinator and university supervisors	To track and record trainees’ progress during training; Measure maintenance or drift in skills; Inform any needed adjustments to training Activities; Evaluation of trainees to confirm minimum practicum competency levels are met; Compare pre- and post-assessments to examine effectiveness of training program; Inform remediation needs and activities; Highlight supervision needs; Inform promotion of trainee for further training as interns.	
**5**	Before Internship	*Functional* 1-Clinical Assessment, 2-Case conceptualization 3-Intervention 4-Supervision	*Modality*: Standardized role plays with mock group members *Formats*: Live observations, video recordings, audio recordings, transcripts	Students (peers)	Students, University rators and Supervisors	Students, Internship coordinator and university supervisors	To track and record trainees’ progress during training; Measure maintenance or drift in skills; Inform any needed adjustments to training Activities; Evaluation of trainees to confirm minimum practicum competency levels are met; Compare pre- and post-assessments to examine effectiveness of training program; Inform remediation needs and activities; Highlight supervision needs; Inform promotion of trainee for further training as interns.	
**6**	End of internship	*Functional* 1-Clinical Assessment, 2-Case conceptualization 3-Intervention 4-Supervision	*Modality*: Standardized role plays with mock group members *Formats*: Live observations, video recordings, audio recordings, transcripts	Students (peers)	Students and University Supervisors	Students, Internship coordinator and university supervisors	Measure maintenance or drift in skills; Evaluation of trainees to confirm minimum practicum competency levels are met; Compare pre- and post-assessments to examine effectiveness of training program; Inform remediation needs and activities; Determine trainee readiness to work in real world; base for certification.	

The initial clinical exposure assessment will record a baseline of competency so that trainees’ progress and performance can be tracked across training and enable supervisors to plan appropriately for training during the exposure. The assessment after the initial clinical exposure will confirm the competency levels that trainees were able to achieve during the exposure and enable comparison between pre-and post-exposure assessments to determine the effectiveness of the first clinical component of the curriculum. Later in the training program, pre-practicum assessment results will be used to determine baseline functional competency of trainees and help supervisors focus on appropriate skill development during practicum. Post-practicum assessment will confirm minimum competency achieved during the practicum and enable comparison of pre-and post-practicum assessments to determine effectiveness of the training program. Further, the results will enable practicum and clinical supervisors to identify and focus skill development during internship. Pre-internship assessment aims at tracking progress in order to determine remediation or refocusing on skills partially or not yet attained because this is the last opportunity before graduation to have clinical encounters and achieve minimum competency levels. Post-internship assessment will seek to evaluate trainees to confirm minimum competency levels met, and make comparison between pre-and post-assessments to determine effectiveness of internship training program and inform any remediation needs and activities. Importantly, the assessment will determine trainee readiness for work in the real world and the need for further training and determination of trainee certification.

The training and assessment of competencies will follow professional developmental trajectory (Blackburn et al., 2001; Gonsalvez and Calvert, 2014) to allow for both formative and summative assessments at various points of the professional development of the trainees. Assessment results will be used to improve future training strategies on the part of supervisors and to help students who might be learning slower than the rest to catch up. Additionally, the results will be used to determine the program feasibility and utility in formal education settings.

### Training faculty in competency-based education and competency assessments and giving competency-based feedback

One fundamental requirement for effective implementation of competency-based curriculum is having skilled faculty members and clinical supervisors in competency-based training, assessment skills, and competency-based feedback provision. Faculty need to know and be able to explain what competency-based training is, organize and carry out role-plays, reliably and accurately rate a standardized role play, select, train and assess actors for competency-based training. Additionally, trainers need to be able to train raters and establish standards for rating role-plays during competency-based trainings and give effective feedback to students based on structured competency assessments. The EQUIP Foundational Helping Skills (FHS) Trainer’s Curriculum which is a brief course designed for training, supervision and assessment of common factors in trainees (Kohrt et al., 2015b) will be adapted to include functional competencies (see Table 2) and used in a four-day EQUIP training for faculty (see Table 4).

**Table 4:** Training schedule and objectives for competency-based education using EQUIP training curriculum.

Day	Session	Learning objective
1	(a) What is EQUIP Competency Based Training?	To be able to explain what EQUIP competency-based training is.
	(b) Role-Play Assessments in Competency Based Training	To be able to explain what EQUIP competency-based training is.
2	(a) Implementing Role Play Assessments	To be able to reliably and accurately rate a standardized role play.
	(b) Training Actors	To be able to select, train and assess an actor for competency-based training.
3	(a) Training Raters	To be able to train raptors and establish benchmarks for rating role-plays during competency-based trainings.
	(b) Feedback in Competency-Based Training	To be able to give effective feedback to students based on structured competency assessments.
4	How to turn curriculum into Competency-Based Training	To be able to effectively implement competency-based training and assessment.

### Pilot testing and evaluation of the competency-based education strategy with faculty and students

Following training of faculty, a pilot implementation of competency-based training will be conducted with students at the School of Psychology at Makerere University. This is done to help improve the instruments and processes before full rollout (Gross, 2006). Results of the study will inform if the feasibility and utility criteria of the EQUIP instruments and procedures were met and identify the need to modify the instruments or method of administration in preparation for the full rollout of the training. The objectives of the pilot study are to (a) Determine the feasibility of EQUIP resources in training students to acquire functional psychology competencies, and (b) Establish the utility of the EQUIP resource for training students in high-intensity psychological interventions in formal pre-service training settings.

Qualitative and quantitative methods will be employed to evaluate the training. Interviews with faculty and site supervisors, both pre- and post-test will be carried out. The results will be augmented with information obtained from students through administration of EQUIP tools and questionnaires. Qualitative interviews will be conducted with faculty pre- and post-training on EQUIP. Interviews with site supervisors will be conducted prior to student placement and 2 months later after student activities on site are completed. Students will complete standardized role-plays pre- and post-clinical placement. The expected outcome of the study will be the determination of the viability and effectiveness of using EQUIP resource to train, supervise and assess foundational and functional professional psychology competencies in pre-service institutions of learning.

### Modify and implement the competency-based education strategy based on pilot results

The pilot study results will be used to change training, supervision and assessments strategies, e.g., which competencies to focus on, what method of training is effective, how long the training should last, what additional preparation the faculty need, how to improve assessment methods, how to improve supervision methods, and how to improve evaluation of competencies.

### Implement ongoing evaluation of the competency-based curriculum with continuous quality improvement

The EQUIP protocol for training, supervising and assessing competency incorporates frequent assessment, tracking of student progression, and enabling student-centered approach to training, to optimize learning and customize curricular elements for each student. Successful implementation will necessitate approval by Makerere University Ethics Committee for compliance with ethical and regulatory issues relating to implementing a developed curriculum.

## Changes in the current curriculum

The developed curriculum will reflect changes in content, method of teaching, assessment and supervision during the implementation of the curriculum, particularly during practicum and internship. The practical course units will have clearly identified competencies and will employ standardized methods of training, assessment and supervision following the EQUIP protocol. Role-play based competency assessments will be conducted throughout the training to monitor progress, to determine minimum competency, and to ensure that the trainees do not engage in harmful behaviors (Kohrt et al., 2018). Additionally, attainment of competence is judged by predetermined output indicators (e.g., number of basic helping skills demonstrated) and not by measures of input (e.g., number of classroom didactic hours completed). Clear methods and prior expected output give a better and easier angle to supervision of competencies (Kavanagh et al., 2003; Watts et al., 2021). The education will additionally follow a modular format, to allow tailoring training to trainees’ needs (Gross, 2006). Finally, grading and the end of training certificate award will both depend on the achievement of the specified competencies rather than on hours completed during practicum and internship.

## Anticipated challenges

There is need train faculty in order to be able to appreciate and embrace the competency-based curriculum and effectively use it in training, assessment and supervision. This involves attitude change. Additionally, there is only one major placement site (Butabika National Mental Referral Hospital) in Uganda which offers appropriate training facility for the students. It is anticipated that the quality of supervision at the placement site might be compromised as many students from other training institutions also use the same facility for mental-health training. Moreover, the staff is numerically limited. Discussion with the staff at the placement site will explore possibilities of better helping the students on practicum through use of group supervision for example. In the long term it is hoped that a Trauma and Stress Center at the School of Psychology Makerere University will be established and will have among other facilities, a mental-health teaching hospital that will cater for the practicum and internship needs of the students. Limited accessibility to eLearning materials and devices (e.g., computers), makes accessing online EQUIP platform learning difficult. Additionally, the cost of using internet is quite high in addition to inconsistent and low-quality internet supply, coupled with intermittent power outages. To offset the problem of accessibility of information resources related to competency-based training and assessment, hard copies of training materials could be made available to the trainers and students.

## Conclusion

The development of a competency-based curriculum for training psychology professionals in formal pre-service training institutions aims to meet an indispensable need of ensuring that the growing workforce of health care professionals are competent, not only in foundational skills but importantly also in functional helping skills. This will hopefully lead to improved quality of care and will be a step in the right direction to achieving the goal of a competent health workforce as outlined in WHO’s Universal Health Coverage goals, and thus contribute to the ideal of equity in mental health care for all people worldwide.

## Data availability statement

The original contributions presented in the study are included in the article/Supplementary material, further inquiries can be directed to the corresponding author.

## Author contributions

The authors made equal, substantial, and direct contribution to the work in the conception, design, draft, and revision of the final version of the manuscript, and approved it for publication.

## Funding

This study was supported by the Fulbright Program (exchange visitor program number G-1-00005), sponsored by the U.S. Department of State’s Bureau of Educational and Cultural Affairs administered by the Institute of International Education (IIE). BA is a recipient of Fulbright African Research Scholar grant, 2021.

## Conflict of interest

The authors declare that the research was conducted in the absence of any commercial or financial relationships that could be construed as a potential conflict of interest.

## Publisher’s note

All claims expressed in this article are solely those of the authors and do not necessarily represent those of their affiliated organizations, or those of the publisher, the editors and the reviewers. Any product that may be evaluated in this article, or claim that may be made by its manufacturer, is not guaranteed or endorsed by the publisher.

## Supplementary material

The Supplementary material for this article can be found online at: https://www.frontiersin.org/articles/10.3389/fpsyg.2022.924683/full#supplementary-material
