# Supplementary material for: Competency-based pre-service education for clinical psychology training in low- and middle-income countries: Case study of Makerere University in Uganda
Source: Front Psychol. 2022 Oct 10;13:924683. doi: 10.3389/fpsyg.2022.924683 (PMC9589034; doi:10.3389/fpsyg.2022.924683)
Supplement: Supplementary file 1 [file Table_1.DOCX]

# SUPPLEMENTARY MATERIALS

***Table 1***: Current Masters of Clinical Psychology Curriculum

| **Year 1** | **Didactic Course:** | **Sep** | **Oct** | **Nov** | **Dec** | **Jan** | | **Feb** | | **Mar** | | **Apr** | | **May** | | **Jun** | | **Jul** | | **Aug** | | **C.U.** | |
| --- | --- | --- | --- | --- | --- | --- | --- | --- | --- | --- | --- | --- | --- | --- | --- | --- | --- | --- | --- | --- | --- | --- | --- |
| ***Sem. 1*** | Professional and Ethical Studies |  |  |  |  |  | |  | |  | |  | |  | |  | |  | |  | | 3 | |
|  | Theories of psychotherapy |  |  |  |  |  | |  | |  | |  | |  | |  | |  | |  | | 3 | |
|  | Adult psychopathology & phenomenology I |  |  |  |  |  | |  | |  | |  | |  | |  | |  | |  | | 3 | |
|  | Neuropsychology |  |  |  |  |  | |  | |  | |  | |  | |  | |  | |  | | 3 | |
|  | Clinical Psychology Technique & practice |  |  |  |  |  | |  | |  | |  | |  | |  | |  | |  | | 2 | |
|  |  | Practicum exposure | | | | |  | |  | |  | |  | |  | |  | |  | |  | | 2 |
|  | **End of Semester 1 Examinations** | | | | | | | | | | | | | | | | | | | | | |  |
| ***Sem. 2*** | Child psychopathology and phenomenology |  |  |  |  |  | |  | |  | |  | |  | |  | |  | |  | | 3 | |
|  | Adult psychopathology and phenomenology II |  |  |  |  |  | |  | |  | |  | |  | |  | |  | |  | | 3 | |
|  | Family and Group Psychotherapy |  |  |  |  |  | |  | |  | |  | |  | |  | |  | |  | | 3 | |
|  | Child and adolescent psychotherapy |  |  |  |  |  | |  | |  | |  | |  | |  | |  | |  | | 3 | |
|  | Child assessment |  |  |  |  |  | |  | |  | |  | |  | |  | |  | |  | | 3 | |
|  | Research Workshop |  |  |  |  |  | |  | |  | |  | |  | |  | |  | |  | | 2 | |
|  | **End of Semester 2 Examinations** | | | | | | | | | | | | | | | | | | | | | |  |
| **Recess *Sem*.** | Research Methodology and Academic Writing |  |  |  |  |  | |  | |  | |  | |  | |  | |  | |  | | 2 | |
|  |  |  |  |  |  |  | |  | |  | |  | | Child Practicum | | | | | | | | 9 | |
|  | Proposal Presentation/ Case write Up |  |  |  |  |  | |  | |  | |  | |  | |  | |  | |  | | 4 | |
| **Year 2** |  |  |  |  |  |  | |  | |  | |  | |  | |  | |  | |  | |  | |
| ***Sem. 1*** | Psychotherapy Technique and Practice (Adult) |  |  |  |  |  | |  | |  | |  | |  | |  | |  | |  | | 2 | |
|  | Health Psychology |  |  |  |  |  | |  | |  | |  | |  | |  | |  | |  | | 3 | |
|  | Adult Assessment |  |  |  |  |  | |  | |  | |  | |  | |  | |  | |  | | 3 | |
|  |  | Adult Practicum | | | | |  | |  | |  | |  | |  | |  | |  | |  | | 6 |
|  | Dissertation/ Case write Up |  |  |  |  |  | |  | |  | |  | |  | |  | |  | |  | | 4 | |
|  | **End of Semester 3 Examinations** | | | | | | | | | | | | | | | | | | | | | |  |
| ***Sem. 2*** | Community Interventions |  |  |  |  |  | |  | |  | |  | |  | |  | |  | |  | | 9 | |
|  |  |  |  |  |  | Internship | | | | | | | |  | |  | |  | |  | | 5 | |
|  | Dissertation/ Case write Up |  |  |  |  |  | |  | |  | |  | |  | |  | |  | |  | | 4 | |
| ***Electives*** | Psychosocial Care of the Dying and Bereaved |  |  |  |  |  | |  | |  | |  | |  | |  | |  | |  | | 3 | |
|  | Gender Issues In Psychotherapy |  |  |  |  |  | |  | |  | |  | |  | |  | |  | |  | | 3 | |
|  | **End of Semester 4 Examinations** | | | | | | | | | | | | | | | | | | | | | |  |
